# Mechanical competence of ovariectomy-induced compromised bone after single or combined treatment with high-frequency loading and bisphosphonates

**DOI:** 10.1038/srep10795

**Published:** 2015-06-01

**Authors:** Camargos G. V., Bhattacharya P., van Lenthe G. H., Del Bel Cury A. A., Naert I., Duyck J., Vandamme K.

**Affiliations:** 1 Department of Oral Health Sciences, BIOMAT Research Cluster & Prosthetic Dentistry, KU Leuven & University Hospitals Leuven, Leuven, Belgium; 2 Biomechanics Section, Department of Mechanical Engineering, KU Leuven, Leuven, Belgium; 3 Department of Prosthodontics and Periodontology, Piracicaba Dental School, University of Campinas, Piracicaba, São Paulo, Brazil

## Abstract

Osteoporosis leads to increased bone fragility, thus effective approaches enhancing bone strength are needed. Hence, this study investigated the effect of single or combined application of high-frequency (HF) loading through whole body vibration (WBV) and alendronate (ALN) on the mechanical competence of ovariectomy-induced osteoporotic bone. Thirty-four female Wistar rats were ovariectomized (OVX) or sham-operated (shOVX) and divided into five groups: shOVX, OVX-shWBV, OVX-WBV, ALN-shWBV and ALN-WBV. (Sham)WBV loading was applied for 10 min/day (130 to 150 Hz at 0.3*g*) for 14 days and ALN at 2 mg/kg/dose was administered 3x/week. Finite element analysis based on micro-CT was employed to assess bone biomechanical properties, relative to bone micro-structural parameters. HF loading application to OVX resulted in an enlarged cortex, but it was not able to improve the biomechanical properties. ALN prevented trabecular bone deterioration and increased bone stiffness and bone strength of OVX bone. Finally, the combination of ALN with HF resulted in an increased cortical thickness in OVX rats when compared to single treatments. Compared to HF loading, ALN treatment is preferred for improving the compromised mechanical competence of OVX bone. In addition, the association of ALN with HF loading results in an additive effect on the cortical thickness.

Osteoporosis is a skeletal disease characterized by low bone mass and micro-architectural deterioration of the bone tissue, leading to decreased bone strength predisposing increased risk of fractures^1^. This disease is associated with estrogen deficiency after menopause, which is responsible for shifting the balance between bone resorption and bone formation toward an increased level of bone resorption^2^,^3^. With an increasing aging population worldwide, osteoporosis has become a growing health concern, since the augmented risk of bone fractures has devastating outcomes in terms of mortality, decreased autonomy and healthcare costs^4^. Thus, therapies that increase the bone mechanical competence in medical compromised conditions are required.

The current therapies to prevent bone loss in estrogen-deficient state are via drugs that affect bone metabolism, such as biphosphonates (BPs). The biphosphonate drug alendronate is a potent anti-resorptive agent^5^, with evidenced reduction of fracture risk in postmenopausal women^6^. However, it has some limitations and side effects that affect long-term administration and patient’s adherence^7^,^8^,^9^. Alternatively to the pharmacological treatment with BPs, biomechanical therapies have been proposed to treat osteoporosis due the profound anabolic effects of mechanical loading on bone^10^. In particular, the application of high-frequency (HF) loading via whole body vibration (WBV) has been shown to promote bone formation, and even recover bone loss arising from osteoporotic conditions^11^,^12^.

The anti-fracture efficacy of these osteoporosis therapies was assessed in previous studies by using dual energy X-ray absorptiometry (DXA) based on bone mineral density (BMD)^13^,^14^. However, DXA-BMD is not an appropriate predictor for the strength of the bone because of the poor association between BMD change and bone fragility^15^. As the method is based on a two-dimensional projection measurement of the three-dimensional bone structure, it lacks the ability to explore macro- and micro-architectural features of the bone^16^. Thus, fracture risk cannot be directly inferred from BMD, and surrogates for assessment of bone mechanical competence that consider bone geometric aspects are needed.

In this context, finite element (FE) analysis provides an approach to assess bone biomechanical properties^17^,^18^, particularly when combined with specimen-specific high-resolution data obtained from micro-computed tomography, from which the bone micro-architecture can be easily obtained^19^. Therefore, the aims of the present study were: (i) to investigate the effect of single or combined application of HF WBV and ALN on the mechanical competence of bone originating from osteoporotic animals; and (ii) to determine how the changes in trabecular and cortical bone micro-structure after these interventions contribute to the bone biomechanical properties.

## Methods

### Animals and experimental design

The protocol of the animal experiment was approved by the local ethical committee (P050/2011), complied with ARRIVE guidelines^20^ for preclinical studies and was performed according to the Belgian animal welfare regulations and guidelines.

A total of 34 female Wistar rats at 12 weeks of age were used in the present study. Twenty-seven animals underwent ovariectomy surgery [OVX], while the remaining seven animals were subjected to sham-ovariectomy surgery [shOVX]. (Sham)-ovariectomy surgery was performed at Charles River Laboratories (Charles River, L’ Arbresle, France). For the rats subjected to sham surgery, the bilateral ovaries were lifted up and returned to their original position, while for the ovariectomized rats, the ovaries were removed. Rats arrived 5 days post-(sham)OVX surgery, with a body weight ranging between 220 g and 250 g. Pair-feeding regimen was initiated immediately in an attempt to control the body weight changes throughout the study. The average daily food consumption of the shOVX animals was determined, and the quantified amount was then provided to the other animals. Animals were weighed at the start and once a week during the study. The OVX group was divided into 2 groups, an untreated group [OVX] (n = 13) and a group treated with the anti-resorptive bisphosphonate drug alendronate [ALN] (n = 14). Alendronate sodium trihydrate (A4978-100MG, Sigma-Aldrich, Bornem, Belgium) was injected subcutaneously 3 days/week at a dose of 2 mg/kg body weight/dose, starting 5 days post-OVX surgery^21^. Saline administration (0.9% NaCl) was performed according to the same time schedule to the rats of the OVX and shOVX groups. Injections were administered till the day of euthanasia (i.e. 14 days after starting the mechanical stimulation). The animals of OVX and ALN groups were further divided into subgroups relative to the loading condition (sham-WBV *versus* WBV), resulting in 4 experimental groups (OVX-shWBV, n = 7; OVX-WBV, n = 6; ALN-shWBV, n = 6; ALN-WBV, n = 8). The 5^th^ group, namely the shOVX group (n = 7), did not receive mechanical stimulation, and served as a negative control to illustrate normal bone characteristics over the course of the experiment. The OVX-shWBV group served as positive control to demonstrate the changes occuring during the development of osteoporotic condition following ovariectomy.

### Vibration device and loading protocol

HF mechanical loading was initiated 6 weeks post-(sham)ovariectomy. This time lapse was considered to be adequate for inducing significant bone changes in the rat long bones in response to ovariectomy^12^. The loading was applied by means of WBV via a custom-made vibration device. The WBV loading was administered during 14 days for 10 minutes/day according to a protocol that consisted of 10 consecutive frequency steps (130, 135, 140, 145, 150, 130, 135, 140, 145 and 150 Hz), each of these applied for 1 minute at an acceleration of 0.3*g*^21^. WBV was applied to the animals individually, taking into account the animal’s body weight. Intervals of 24-hours between the loading sessions were respected.

### Specimen preparation and micro-X-ray computed tomography analysis

After euthanasia of the animals by cervical displacement under isoflurane-induced anaesthesia, the hindlimbs were excised and the surrounding soft tissues removed. The tibiae were retrieved and immediately fixated in 10% CaCO_3_–buffered formalin solution (pH 7.4) at 4 °C for 48 h. The samples were further kept in the 70% ethanol at 4 °C until the day of μCT scanning.

For assessment of the bone micro-architecture in the experimental groups, the proximal part of the tibiae was examined *ex vivo* using a desktop μCT system, commercially available as Skyscan 1172 (Bruker, Kontich, Belgium). During scanning, the tibia was placed in the polyethylene tube and immobilized inside the tubes by means of soft modeling clay. The bone samples were scanned along the mid-sagittal planes in the mid-diaphyseal regions to obtain the μCT images. The scanning parameters were 6 μm pixel size, 50 kV X-ray voltage, 200 μA electric current and 0.5 mm Al filter.

**Figure 1:**
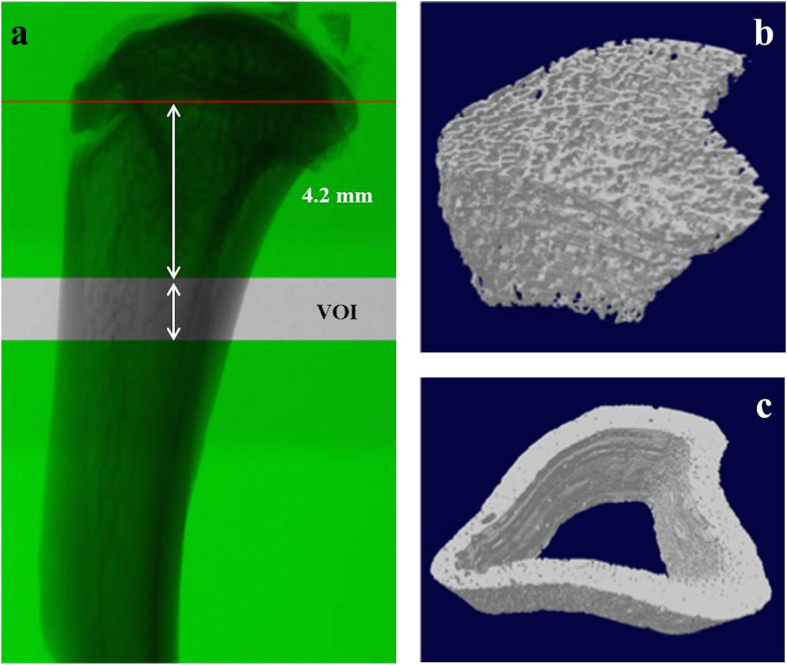
Representative μCT images of rat tibia. **(a)** VOI selected for trabecular and cortical bone analysis: 4.2 mm distally from the growth plate level and extending towards the distal diaphysis over a distance of 1.5 mm; (b) 3D view of trabecular (b) and cortical bone (c) volume reconstructed from the selected ROI.

Subsequently, the reconstructed 3D data sets were quantified using CTAn automated image analysis system (Bruker, Kontich, Belgium). For this, the volume of interest (VOI) for both trabecular and cortical analyses was defined in axial direction by using the growth plate as reference. The VOI started at a distance of 4.2 mm distally from the growth plate and extended towards the diaphysis for 1.5 mm (250 slices) (Fig. 1a). In each transverse slice of the VOI, the ROI was delineated manually matching with the area occupied by trabecular (Fig. 1b) or cortical bone (Fig. 1c) to perform trabecular or cortical bone analysis, respectively.

For trabecular bone analysis, the bone volume (BV), bone volume fraction (BV/TV), trabecular thickness (Tb.Th), trabecular separation (Tb.Sp) and trabecular number (Tb.N) were calculated 3D as measurements of trabecular bone mass and its distribution. The trabecular architecture was quantified by calculating the connectivity of the trabecular network (trabecular bone pattern formation, TBPf) and structure model index (SMI). For cortical bone analysis, the periosteal perimeter (Ps.Pm), medullary area (Ma.Ar), endocortical perimeter (Ec.Pm) and mean polar moment of inertia (MMI) were calculated in 2D as measurements of cortical bone mass and its distribution. The porosity (Ct.Po) and cortical thickness (Ct.Th) were calculated 3D as measurements of cortical bone mass and its distribution according to standard procedures^22^.

### Structural analysis

**Figure 2:**
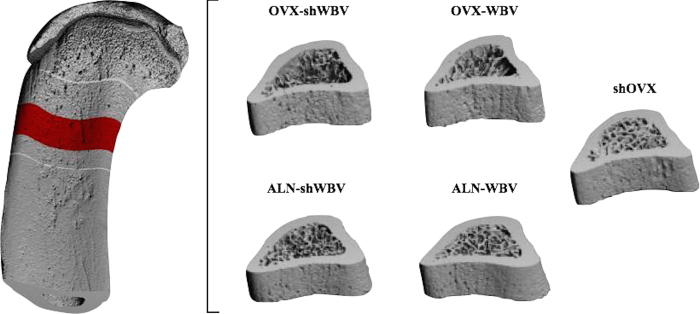
Representative three-dimensional models of the tibial diaphysis for each group (shOVX, OVX-shWBV, OVX-WBV, ALN-shWBV and ALN-WBV) used in μFE analysis.

Finite element models of all bone samples were created by a direct conversion of bone voxels to cubic hexahedral elements^23^. The three-dimensional (3D) models of bone presented a fixed length in z-direction (3.0 mm) that corresponded to the VOI evaluated in the μCT analysis (Fig. 2). These models consisted of 3.8 to 6.4 million elements, having between 12.2 and 21.7 million degrees of freedom. Linear and isotropic material behavior was assumed. Material properties were applied, using typical values for bone (Young’s modulus, E = 10 GPa and Poisson’s ratio, ν = 0.3). Identical material properties were used for all groups, thereby enabling to isolate the effects of micro-architectural differences on bone stiffness and bone strength. Boundary conditions that represented uniaxial compression tests were defined in order to determine the bone mechanical competence. The nodes at the top surface of samples were displaced in axial direction by 1% of total height. Since all models had the same z-extent, 1% of strain in z-direction corresponds to a standard z-displacement of 0.03 mm. All nodes at the bottom of the models were restrained in the direction of the loading, except for two nodes that were restricted in the transversal plane too, in order to prevent rigid body rotation and translations.

For the solution of these models with up to 20 million degrees of freedom, a parallel linear finite element package (ParFE) with an algebraic multigrid preconditioner was used^24^. All the calculations were performed at the National Supercomputing Center (CSCS, ETH Zürich, Switzerland), where the code was run on up to 3072 cores of a CRAY XT5™. The latter device consists of 3688 AMD hexa-core Opteron processors clocked at 2.4 GHz and a high-speed interconnect with a bandwidth of 9.6 GB/s and a latency of 5 μs. Numerical post-processing was done on a Sun Fire™ system; for visualization, the parallel open source program ParaView^25^ was used, running on an HP-XC cluster. Bone stiffness [N/mm] was determined from the FE model as the slope of the linear force–displacement relationship obtained up to 1% apparent compressive strain. Bone strength was determined as the minimum force required for 2% of the voxels (representing 2% of total bone volume) reaching at least 0.7% effective strain^26^,^27^.

### Statistical analysis

Results of the bone micro-structural and biomechanical parameters were expressed as means ± standard deviation (SD). The differences between the means for the experimental groups were compared using one-way analysis of variance (ANOVA). Multiple comparisons between the groups were performed using Scott–Knott method and differences were considered significant at *p* < 0.05.

## Results

### Rats body weight

The body weight recordings at the day of arrival of the animals (*i.e.* 5 days post-(sh)OVX surgery) revealed a slightly higher body weight for OVX and ALN animals compared to shOVX animals (246.2 ± 14.6 g for OVX, 241.1 ± 13.9 g for ALN and 230.9 ± 14.1 g for shOVX). As a consequence of ovariectomy surgery, the animals from OVX and ALN group indeed have the tendency to gain weight compared to shOVX group, despite of the pair-feeding regime (272.5 ± 17.7 g for OVX, 268.7 ± 18.9 g for ALN and 233.0 ± 12.8 g for shOVX). However, HF WBV application did not affect the rats’ body weight changes in all experimental groups.

### ANOVA results

ANOVA revealed a statistically significant difference between the groups (shOVX, OVX-shWBV, OVX-WBV, ALN-shWBV, ALN-WBV) for bone stiffness (*p* = 0.000), bone strength (*p* = 0.000), trabecular micro-structural parameters (BV, *p* = 0.000; BV/TV, *p* = 0.000; Tb.Th, *p* = 0.014; Tb.SP, *p* = 0.000; Tb.N, *p* = 0.000; TBPf, *p* = 0.000; SMI, *p* = 0.000) and cortical micro-structural parameters (Ct.Th, *p* = 0.001; Ct.Po, *p* = 0.011; Ps.Pm, *p* = 0.004; Ma.Ar, *p* = 0.012; and MMI, *p* = 0.001), except for Ec.Pm (*p* = 0.056). The results of the multiple comparions between all experimental groups for each biomechanical and micro-structural parameter are shown in Tables 1 and 2.

### Effect of WBV and/or ALN on bone micro-structure

OVX caused a marked deterioration of the trabecular and cortical bone micro-structure, manifested as a considerable decrease in Ct.Th (−9%), BV (−52.6%), BV/TV (−63.6%), Tb.N (−75.0%) and increase in Ma.Ar (+21.9%), Tb.Sp (+163.4%), TBPf (+466.7%) and SMI (+144.4%) (OVX-shWBV *versus* shOVX; Tables 1 and 2). Simultaneously, as a compensatory mechanism after ovariectomy, a slight increase in Ps.Pm was observed in OVX-shWBV compared to shOVX (+2.8%, Table 2). Mechanical stimulation of OVX rats with HF WBV for 14 days had no effect on the trabecular bone, except for TBPf (+23.5%, OVX-WBV *versus* OVX-shWBV; Table 1). At cortical level, the mechanical treatment was able to partially reverse the bone micro-structural deterioration after ovariectomy by decreasing substantially the Ma.Ar (-20.4%) and increasing Ct.Th (+7.2%) (OVX-WBV *versus* OVX-shWBV). Additionally, it is noteworthy that there were no differences between the cortical micro-structural parameters quantified for OVX-WBV and shOVX (table 2).

Unlike the single HF loading treatment, the pharmacological treatment with alendronate revealed a positive effect mainly on the trabecular bone micro-architecture of OVX rats, which was demonstrated by an increase in BV (+107.8%), BV/TV (+120%) and Tb.N (+300%) and a decrease in Tb.Sp (−58.2%), TBPf (−64.7%) and SMI (−50.0%). However, ALN administration was not able to improve the Tb.Th after ovariectomy (ALN-shWBV *versus* OVX-shWBV, Table 1). At cortical level, ALN administration was associated with an increased Ct.Th (+9.8%) and Ct.Po (+60.8%) compared to OVX-shWBV (ALN-shWBV *versus* OVX-shWBV, Table 2). When compared to shOVX group, ALN also presented higher values of Ct.Po (+38.8%), Ps.Pm (+5%), MMI (+18.5%) and Ma.Ar (+20.3%) (ALN-shWBV *versus* shOVX, Table 2). Finally, the combined application of ALN and HF resulted in a greater increase in Ct.Th when compared to shOVX or ALN-shWBV groups (+7.1% for ALN-WBV *versus* shOVX and +7.4% for ALN-WBV *versus* ALN-shWBV; Table 2).

### Effect of WBV and/or ALN on bone mechanical competence

OVX caused a substantial decrease in bone stiffness (−10.7%) and bone strength (−10.4%) compared to shOVX (OVX-shWBV *versus* shOVX; Tables 1 and 2). The mechanical treatment of OVX rats with HF loading via WBV (OVX-WBV) did not affect bone stiffness nor strength when compared to OVX-shWBV. However, the pharmacologic treatment with ALN, combined or not with WBV, significantly increased bone stiffness (+15.8% for ALN-shWBV and 14.5% for ALN-WBV) and bone strength (+13.8% for ALN-shWBV and +17.6% for ALN-WBV) compared to the respective untreated OVX condition, with values equalling those of the shOVX group. In addition, no significant differences were detected between ALN-shWBV and ALN-WBV groups regarding bone stiffness and bone strength (Tables 1 and 2).

## Discussion

The higher resolution of micro-computed tomography images and mechanical testing procedures^28^,^29^ has highlighted the significance of several bone properties other than BMD for bone strength^30^, which can improve the fracture risk prediction and the assessment of anti-fracture efficacy of osteoporosis therapies. Impaired bone strength associated with altered bone turnover might result from decreases in the amount of bone mass, changes in bone micro-architecture or geometry, in the biophysical properties of bone tissue, or even from a combination of all of the above^30^. Therefore, the present study by using three-dimensional analysis, μCT and μFE analyses, evaluated the potential relation of bone mechanical competence with bone micro-structural changes of ovariectomy-induced compromised bone after single or combined treatment with HF loading and bisphosphonates.

In OVX osteoporotic rats, a decreased bone mechanical competence associated with a cortex thinning and trabecular bone loss was observed. This increased bone fragility is a result of the negative bone multicelullar unit balance induced by estrogen withdrawal after ovariectomy, which is the morphological basis of bone loss and structural deterioration^31^. With a larger surface-to volume ratio, trabecular bone is rapidly affected by increases in bone resorption^32^. Individual trabeculae become progressively thinner, shifting from a plate-like shape to a rod-like shape while trabecular separation increases. Progressive perforation of individual rods leads to the loss of trabecular connectivity and reduces the number of trabeculae, resulting in trabecular micro-architecture deterioration^33^. These changes in trabecular micro-architecture were also observed in previous studies with ovariectomized rats and rapidly compromise bone strength^34^,^35^. At cortical level, a higher endocortical resorption was noticed in OVX animals represented by an increase in Ma.Ar compared to shOVX, leading to a reduction in bone mass and cortical thickness despite of the slight periosteal expansion after ovariectomy. Clinical studies in postmenopausal women also reported about 50% of cortical bone loss as a result of remodelling within the inner (endosteal) surface adjacent to the marrow by cavitation^36^. Weakening of the cortical bone compartment is also of major importance for fragility fractures once cortical bone represents a substantial amount of the total bone mass, especially in the appendicular skeleton^36^. These results therefore demonstrate that micro-architectural changes after ovariectomy in either cortical or trabecular bone are responsible for significant variations in the bone mechanical properties.

The mechanical treatment with HF loading was not able to oppose the negative effects of ovariectomy on the bone mechanical competence. HF loading increased the cortical thickness of OVX rats by decreasing the endocortical resorption, represented in this study by lower values of Ma.Ar compared to OVX-shWBV. Notwithstanding this, the increase in cortical thickness after HF loading was not able to improve the OVX bone strength and bone stiffness since this mechanical stimuli did not prevent the trabecular bone deterioration, as also demonstred by Hatori *et al.*^37^. In agreement with our results, a recent study with ovariectomized rats also did not find substantial effects of mechanical stimulation neither on bone biomechanical properties nor on trabecular bone micro-architecture (4 weeks of WBV loading)^38^. However, after 12 weeks of mechanical treatment an improvement of the trabecular bone parameters of OVX animals could be noticed^38^. These results are in contrast to those reported by Tezval *et al.*^35^ and Sehmisch *et al.*^34^, who found that treatment with WBV for 5 weeks resulted in improved bone strength and bone mass, equalling the levels as observed in untreated sham rats. It is worth noting that differences could arise due to the skeletal site evaluated in the latter studies. The present study as well as the study by Chen and co-workers^38^ investigated the effects of mechanical treatment at the tibia, while the study of Tezval *et al.*^35^ and Sehmisch *et al.*^34^ evaluated the femur and vertebral body, respectively. The tibia was chosen because this sites enables concomittantly trabecular and cortical bone analysis, which is not the case for the vertebral body. Furthermore, the femura were used for isolation of bone-marrow derived stromal cells and investigation of the role of hormonal and mechanical influences on the progenitor cells’ biology. Also, the response of the skeletal tissues to mechanical loading depends on several factors including magnitude, duration and rate of stimulus^39^. Hence, it might be that the short duration of experimental period used in the present study, namely 14 days, has been insufficient to increase the bone mechanical competence in ovariectomized rats, since more densely mineralized bone is removed after ovariectomy and replaced by younger less mineralized bone, which has reduced stiffness^40^.

Unlike single HF loading treatment, alendronate administration was able to significantly enhance the bone mechanical competence in ovariectomized rats. This might be attributed to the efficacy of this drug in preventing ovariectomy-induced trabecular bone micro-architectural deteterioration. The large surface area of trabecular bone is an advantage in therapeutics because it facilitates the access of biphosphonates to inhibit matrix remodeling^32^. At the same time, a limited effect of ALN on the endocortical bone remodeling was observed, since the drug was not able to decrease the Ma.Ar enlargement that occurs after ovariectomy. As biphosphonates act by binding avidly to mineralized bone matrix^41^, and considering that at intracortical or endocortical surfaces there is less volume of mineralized cortical bone matrix, the accessibility of this drug to the bone remodeling that is occuring at these surfaces is reduced^42^,^43^. Consequently, osteoclasts initiating remodeling upon a Haversian canal surface might not encounter and engulf matrix containing drug and continue reabsorbing bone at endocortical surface^32^, particularly in rodents that lack a true Haversian canal system^44^. Nevertheless, the association of ALN with HF loading seems to provide an additive effect at the level of endorcortical surface, demonstrated by lower values of Ma.Ar in comparison to the single pharmacological treatment. Thus, the effect of HF stimuli on the endocortical surface combined with the effect of ALN at the periosteal surface level contributed for an enlarged cortical thickness when compared to single application of these treatments. Chen *et al.*^38^ also showed that WBV enhanced the effect of alendronate on the trabecular micro-architecture in ovariectomized rats over 12 weeks, but the micro-structural changes at cortical level after this combined treatment was not investigated. In the present study, although there is a trend of the combined treatment with HF loading and ALN in improving trabecular micro-architecture and bone strength after ovariectomy, the results were statistically not significant when compared to single pharmacological treatment over 2 weeks. Therefore, long-term studies are needed in order to elicit the role of HF loading, combined or not with ALN, on trabecular bone.

From a biomechanical point of view, an effective treatment for bone fragility should improve the extrinsic biomechanical properties of bone but at the same time not substantially impair the intrinsic properties. However, it is rare for a treatment to achieve this combination of effects^45^. Bisphosphonates, by reducing excessively bone turnover, decreases the renewal of bone tissue and consequently increases the accumulation of older and extensively mineralized bone^46^. As a consequence, the bone becomes more brittle and therefore unable to absorb energy by elastic deformation. Additionally, it leads to the accumulation of damaged bone, facilitating micro-crack proliferation^47^. Therefore, recent studies have provided a well documented association between atypical fractures and long-term oral intake of alendronate^8^,^48^, which is a growing clinical concern^9^. Hence, in order to achieve a safer and better clinical effect on osteoporosis treatment, the combined use of ALN with HF WBV could be advised. Exercise–induced improvements in the inorganic bone component does not seem to restrict the mineralization degree, improves also the water content^49^, which is assumed to confer additional biomechanical advantages^50^ as the tissue become less brittle and more able to accomodate the loads applied to the bone before developing micro-damage. This combination therapy might decrease the dosage of ALN in clinical patients. Using a reduced dosage of biphosphonates (lower weekly dose or dosing every 2 weeks) is an unproven but appealing alternative^9^, providing an adequate but less complete surpress of bone turnover^51^ and is likely to provide fracture risk reduction.

The cross-sectional design and the relatively short-term experiment period of this study were the main limitations. Therefore, it remains unclear whether longer-term changes on the bone micro-architecture after HF and ALN interventions would still affect the bone mechanical competence. Furthermore, an *in silico* technique was used in this study to determine the bone stiffness and bone strength based on bone micro-architecture since this methodology was successfuly validated in previous studies showing high precision^17^,^18^. Future studies should also consider the inclusion of other methodologies to evaluate the biophysical properties of bone tissue, such as the degree and type of collagen cross-linking, hydration, the mineral crystal size and their crystallinity in order to provide a complete insight into the bone mechanical competence.

In conclusion, in comparison to single treatment with HF loading, ALN proved to be better in improving the mechanical competence of OVX-induced compromised bone. In addition, the association of ALN with HF loading resulted in an additive anabolic effect on the cortical thickness.

## Additional Information

**How to cite this article**: Camargos, G. V. *et al*. Mechanical competence of ovariectomy-induced compromised bone after single or combined treatment with high-frequency loading and bisphosphonates. *Sci. Rep.* **5**, 10795; doi: 10.1038/srep10795 (2015).

## Figures and Tables

**Table 1:** Results for bone stiffness, bone strength and trabecular bone micro-structural parameters (BV, BV/TV, Tb.Th, Tb.Sp, Tb.N, TBPf, SMI) for each group evaluated.

	Mean ± Standard Deviation								
**Groups**	**Stiffness** (N/mm)	**Strength** (N)	**BV** (μm^3^)	**BV/TV** (%)	**Tb.Th** (μm)	**Tb.Sp** (μm)	**Tb.N** (1/mm)	**TBPf** (1/mm)	**SMI**
shOVX	68280.0 ± 1571.6^**b**^	1042.6 ± 32.1^**b**^	1.9E+09 ± 2.2E+08^**b**^	27.5 ± 2.5^**b**^	66.1 ± 4.5	189.6 ± 8.0^**b**^	4.0 ± 0^**b**^	3.0 ± 3.0^**b**^	0.9 ± 0.3^**b**^
OVX-shWBV	60974.0 ± 3311.8^**a**^	934.2 ± 59.0^**a**^	9.0E+08 ± 3.1E+08^**a**^	10.0 ± 4.7^**a**^	64.6 ± 2.7	499.4 ± 231.3^**a**^	1.0 ± 1.0^**a**^	17.0 ± 4.0^**a**^	2.2 ± 0.3^**a**^
OVX-WBV	58399.9 ± 3989.2^**a**^	900.1 ± 63.5^**a**^	5.3E+08 ± 2.2E+08^**a**^	7.8 ± 3.1^**a**^	65.0 ± 5.4	636.6 ± 250.4^**a**^	1.0 ± 1.0^**a**^	21.0 ± 4.0^**a+b**^	2.6 ± 0.3^**a**^
ALN-shWBV	70596.1 ± 5729.3^**b**^	1063.2 ± 79.8^**b**^	1.9E+09 ± 4.5E+08^**b**^	22.0 ± 3.7^**a+b**^	57.7 ± 2.3^**a+b**^	208.7 ± 22.6^**b**^	4.0 ± 1.0^**b**^	6.0 ± 5.0^**b**^	1.1 ± 0.5^**b**^
ALN-WBV	69834.2 ± 4804.4^**b**^	1098.8 ± 69.8^**b**^	1.8E+09 ± 4.3E+08^**b**^	23.5 ± 3.3^**a+b**^	60.8 ± 6.3^**a+b**^	210.1 ± 13.9^**b**^	4.0 ± 0^**b**^	4.0 ± 1.0^**b**^	1.0 ± 0.2^**b**^

Means ± SD followed by “a” and “b” represent statistically significant difference from the ShOVX and OVX-shWBV group, respectively (p < 0.05).

**Table 2:** Results for bone stiffness, bone strength and cortical bone micro-structural parameters (Ct.Po, Ps.Pm, Ma.Ar, MMI, Ec.PM, Ct.Th) for each group evaluated.

	Mean ± Standard Deviation							
**Groups**	**Stiffness** (N/mm)	**Strength** (N)	**Ct.Po** (%)	**Ps.Pm** (μm)	**Ma.Ar** (μm^2^)	**MMI** (μm^4^)	**Ec.Pm** (μm)	**Ct.Th** (μm)
shOVX	68280.0 ± 1571.6^**b**^	1042.6 ± 32.1^**b**^	1.4 ± 0.6	13557.6 ± 399.3^**b**^	4.8E+06 ± 3.7E+05^**b**^	1.2E+13 ± 1.4E+12	12275.2 ± 704.8	451.6 ± 20.0^**b**^
OVX-shWBV	60974.0 ± 3311.8^**a**^	934.2 ± 59.0^**a**^	1.2 ± 0.4	13944.3 ± 493.8^**a**^	5.9E+06 ± 4.6E+05^**a**^	1.3E+13 ± 1.5E+12	13727.2 ± 1595.5	410.8 ± 25.2^**a**^
OVX-WBV	58399.9 ± 3989.2^**a**^	900.1 ± 63.5^**a**^	1.0 ± 0.2	13265.2 ± 350.6^**b**^	4.7E+06 ± 3.3E+05^**b**^	1.2E+13 ± 1.1E+12	12027.7 ± 462.4	440.7 ± 23.3^**b**^
ALN-shWBV	70596.1 ± 5729.3^**b**^	1063.2 ± 79.8^**b**^	1.9 ± 0.7^**a+b**^	14231.8 ± 428.9^**a**^	5.8E+06 ± 6.3E+05^**a**^	1.5E+13 ± 1.9E+12^**a+b**^	13604.8 ± 940.8	450.4 ± 16.7 ^**b+c**^
ALN-WBV	69834.2 ± 4804.4^**b**^	1098.8 ± 69.8^**b**^	1.9 ± 0.5^**a+b**^	14296.5 ± 726.0^**a**^	5.5E+06 ± 1.2E+06^**a**^	1.6E+13 ± 2.4E+12^**a+b**^	13498.0 ± 1846.6	483.6 ± 41.2 ^**a+b**^

Means ± SD followed by: “a” represents statistically significant difference from ShOVX; “b” represents statistically significant difference from the OVX-shWBV group; and “c” represents statistically significant difference between ALN-shWBV and ALN-WBV (p < 0.05).
